# Answers to Correspondents

**Published:** 1908-02-29

**Authors:** 


### ANSWERS TO CORRESPONDENTS.

"Fucus Vesiculosus."—In reply to two non-medical correspondents, who have written for further particulars of the use of this substance in the reduction of adipose tissue, we would say that fucus vesiculosus, mentioned in an article in The Hospital of December 28, 1907, is not a patent or proprietary article. Every medical man is familiar with it to some extent, and is competent to decide whether it is suitable for particular cases. We advise our correspondents to consult their medical attendants on this point in the first instance. Except as the result of a thorough personal examination it is impossible to say whether an individual case should or should not have this medicine.

